# Evidence of indiscriminate fishing effects in one of the world’s largest inland fisheries

**DOI:** 10.1038/s41598-018-27340-1

**Published:** 2018-06-12

**Authors:** Peng Bun Ngor, Kevin S. McCann, Gaël Grenouillet, Nam So, Bailey C. McMeans, Evan Fraser, Sovan Lek

**Affiliations:** 1 Fisheries Administration, No. 186, Preah Norodom Blvd., Khan Chamcar Morn, P.O. Box 582, Phnom Penh, Cambodia; 2 CNRS, Université Toulouse III Paul Sabatier, ENFA; UMR5174 EDB (Laboratoire Évolution & Diversité Biologique), 118 route de Narbonne, F-31062 Toulouse, France; 3 University of Guelph, Guelph Ontario, Canada; 4 Mekong River Commission Secretariat, Vientiane, Lao PDR; 5 University of Toronto Mississauga, Mississauga, Ontario, Canada

## Abstract

While human impacts like fishing have altered marine food web composition and body size, the status of the world’s important tropical inland fisheries remains largely unknown. Here, we look for signatures of human impacts on the indiscriminately fished Tonle Sap fish community that supports one of the world’s largest freshwater fisheries. By analyzing a 15-year time-series (2000–2015) of fish catches for 116 species obtained from an industrial-scale ‘*Dai’* fishery, we find: (i) 78% of the species exhibited decreasing catches through time; (ii) downward trends in catches occurred primarily in medium to large-bodied species that tend to occupy high trophic levels; (iii) a relatively stable or increasing trend in catches of small-sized species, and; (iv) a decrease in the individual fish weights and lengths for several common species. Because total biomass of the catch has remained remarkably resilient over the last 15 years, the increase in catch of smaller species has compensated for declines in larger species. Our finding of sustained production but altered community composition is consistent with predictions from recent indiscriminate theory, and gives a warning signal to fisheries managers and conservationists that the species-rich Tonle Sap is being affected by heavy indiscriminate fishing pressure.

## Introduction

Globally, inland waters extend over an area of about 7.8 million km^2^ and are among the most biologically productive and diverse ecosystems on earth^1–3^. Inland capture fisheries are important sources of food security, livelihoods, and recreation for tens of millions of people worldwide^4,5^. Overall, inland fisheries employ approximately 61 million people^6^ and represent 11.3% of the world total capture fish production^7^. These fisheries, however, are facing numerous challenges from human activities, namely, population growth, habitat degradation, hydrological changes, pollution, invasive species and climate change^1,8–11^.

Worries over the fate of inland waters^12^, along with the concern that higher trophic levels of marine food webs are being unsustainably exploited, have grown during the last decade. In particular, fisheries ecologists have recently argued that increased indiscriminate fishing pressure is reducing large-sized, slower-growing species with late maturity, and replacing them with smaller-sized, faster-growing species that mature earlier^13–16^. This leads to an overall reduction in the body size and, consequently, a reduction in the overall trophic level of the fish assemblage remaining in an ecosystem. Ultimately, these changes are expected to be reflected in catch composition^12,17–20^. Shifts through time in the slope of the catch-size spectra and decreases in the size of individual fish are also among the key structural and functional ‘signatures’ of indiscriminate fishing on the fish community^21^. Currently, however, much of the fisheries impact research has focused on marine systems and very little is known about freshwater fisheries in the sub-tropical and tropical environments such as the Mekong River Basin^22,23^. What limited evidence exists from inland tropical fisheries suggests declining catches, particularly in Asia and Africa where fish protein is of paramount importance in terms of food security. Hence, there are increasing calls in the literature that inland tropical fisheries should receive more research attention^1,4,5,8^.

This paper contributes to the literature on inland tropical fisheries, demonstrating that larger higher trophic level fish are being depleted in one of the world’s largest freshwater fisheries, while smaller, lower trophic levels organisms are increasing in a manner that sustains overall fish catches. Towards this, we study temporal dynamics of 116 fish species in the Tonle Sap over 15 years. The Tonle Sap, at the whole fishery scale, has been shown to employ an amazing amount of fishing gears applied broadly across habitats and seasons in a manner that uniformly catches a high diversity of fishes (see^13^, Fig. 5). This approach is highly suggestive of a relatively indiscriminate fishery. The dataset for the study was obtained from a standardized biological catch assessment of an industrial-scale ‘*Dai* fishery’ that operates during the dry season in the Tonle Sap River. We explore how temporal trends of fish catch captured by this fishery relate to each species’ maximum body size and trophic level. We also examine changes in the body weight and length of individual fish for select species over the assessment period.

## Results

### Summary of the fishery catch

Over the 15-year assessment period, 141 fish species belonging to 12 orders, 36 families and 93 genera were recorded. The four main orders, representing 90% of the total species counts were: Cypriniformes (59 species), Siluriformes (36), Perciformes (23) and Clupeiformes (7). The rest contained one to three species in each order. Five families forming 95% of the total catch by weight were Cyprinidae (84%), Pangasiidae (4%), Cobitidae (4%), Siluridae (3%) and Cynoglossidae (1%). Three genera forming 66% of the total catch were Henicorhynchus (42%), Paralaubuca (12%), Labiobarbus (12%). Henicorhynchus contained three species namely *Henicorhynchus lobatus* (17%), *Henicorhynchus* sp. (15%) (synonym of *Lobocheilos cryptopogon* and *H*. *cryptopogon)* and *H*. *siamensis* (10%); whereas, Paralaubuca encompassed only one species *Paralaubuca barroni* (synonym of *P*. *typus*), and finally, Labiobarbus consisted of two species: *L*. *lineatus* (10%) and *L*. *siamensis* (2%). By size category, 75% of catch was from species with maximum total length (maxTL) < = 30 cm, 9% with maxTL 31–60 cm, 9% with maxTL 61–90 cm and 6% with maxTL >90 cm. By trophic level, 70% of catch was from species with trophic level < = 2.75, 27% with trophic level = 2.76–3.75 and 3% with trophic level >3.75. Ecologically, 82% of catch was longitudinal (riverine) migratory species, 17% was lateral-migration species, and about 1% is from a combination of estuarine, marine and floodplain resident species. For relative catch weight of 116 species captured by the *Dai* fishery, see Supplemental Information Fig. S4. We also found an overall declining trend in species diversity (evenness index) (see Fig. S5), signifying that fish community was highly unevenly distributed particularly between 2008 and 2015.

### Temporal change in fish catch and relationship with maximum length and trophic level

**Figure 1:**
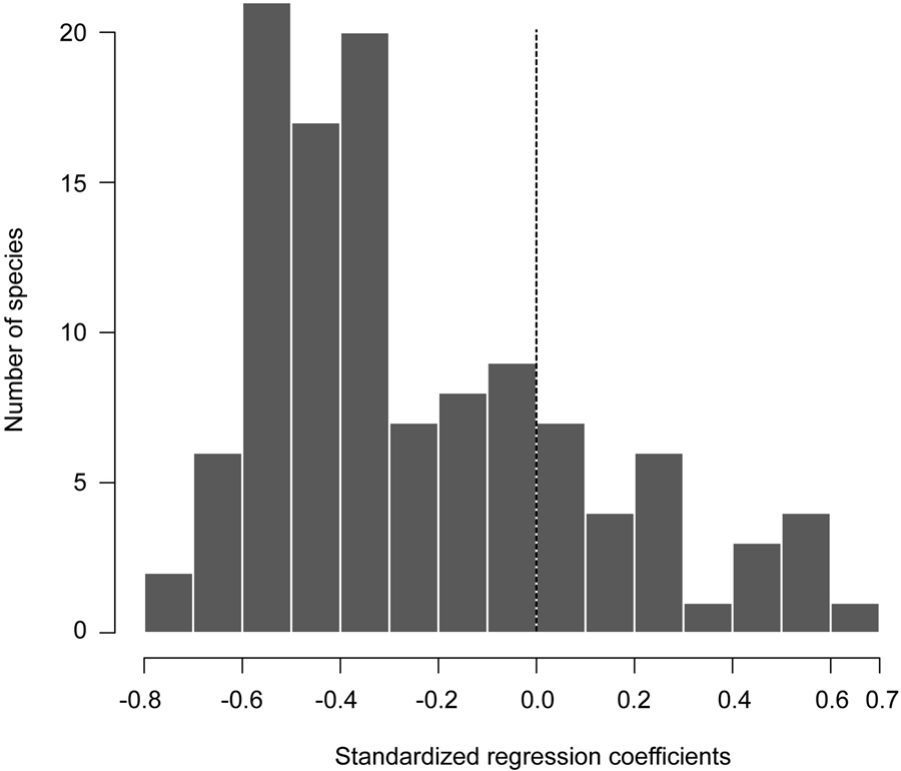
Distribution of standardized regression coefficients of seasonal catches of 116 fish species recorded at the *Dai* fishery, Tonle Sap River from the fishing season of 2000/01 to 2014/15.

The distribution of the standardized regression coefficients for all 116 species, which reflected the nature of the relationship between seasonal fish catch and time for each species, was skewed to the right, centered around −0.4, and spread between −0.78 and 0.66 (Fig. 1). Out of the 116-total species, 90 (78%) had negative standardized regression coefficients. These results indicate that the seasonal catches of these species harvested by the *Dai* fishery declined over the 15 years studied. On the contrary, there were also species (26 out of 116 or 22%) with positive standardized regression coefficients, indicating an increase in the catch of these species by the *Dai* fishery. Interestingly, *Oreochromis mossambicus* is an exotic species that was among the largest positive coefficients observed. In addition, *Labiobarbus lineatus*, *Henicorhynchus lobatus* and *H*. *cryptopogon* (synonym of *Lobocheilos cryptopogon*) are all known to be highly prolific and form the largest proportion of the catch from the fishery. These species also had positive standardized coefficients (see Table S6 for standardized regression coefficients, maxTL and trophic level for each species). In fact, the increase in these species stabilized the seasonal *Dai* catches as it was evidenced in the total catch of the fishery which was stationary over the study period (p-value = 0.982, Fig. S8).

**Figure 2:**
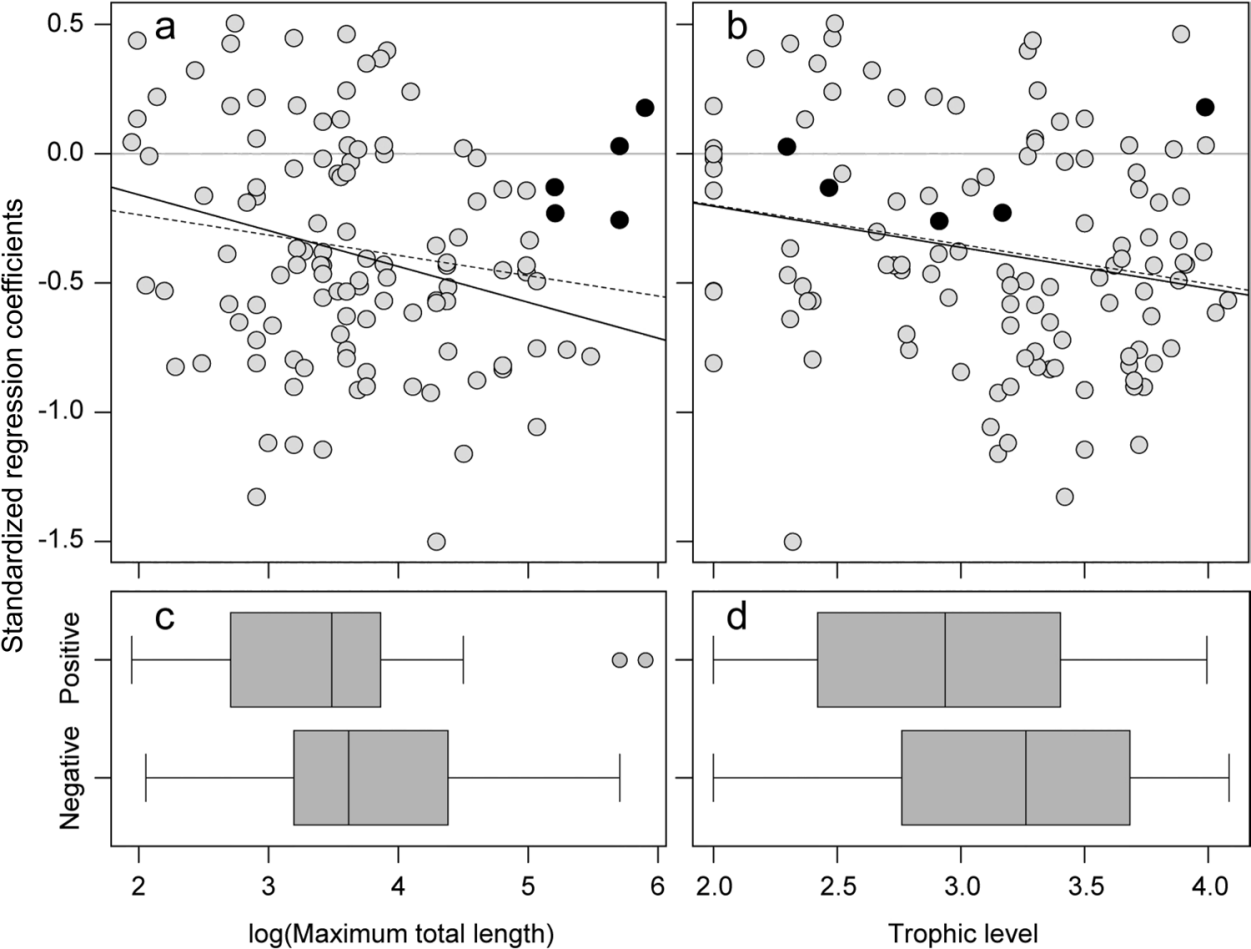
Relationship between (log +1 transformed) standardized regression coefficients of species composition derived from seasonal catches of 116 fish species recorded at the *Dai* fishery in the Tonle Sap River from the fishing season of 2000/01 to 2014/15, and (**a**) their corresponding log-transformed maximum total lengths (maxTL in cm) and (**b**) trophic levels. Solid points represent endangered (en) and critically endangered (ce) species. Dashed lines show linear regression lines to predict the relationships when all species are considered, and solid lines are linear regression lines when en and ce are excluded from (**a**) and (**b**). Model summary (**a**) when all species are included: slope = −0.08, p-value = 0.08, r^2^ = 0.03; and when en and ce are excluded: slope = −0.13, p-value = 0.006, r^2^ = 0.06. Model summary (**b**) when all species are included: slope = −0.15, p-value = 0.02, r^2^ = 0.04; and when en and ce are excluded: slope = −0.16, p-value = 0.02, r^2^ = 0.05. Boxplots show (**c**) distribution of maxTL and (**d**) trophic level for the positive and negative standardized regression coefficient values of all 116 species. Using Mann-Whitney rank sum tests for significant differences, p-values for (c) and (d) are 0.02 and 0.08, respectively.

Species with declining catch in the *Dai* fishery were disproportionately represented by those with larger body sizes and higher trophic levels based on linear regressions (Fig. 2a,b), which demonstrated overall negative relationships between the log +1 transformed standardized regression coefficients and the corresponding log-transformed maxTL (slope = −0.08, p-value = 0.08, r^2^ = 0.03), and trophic level (slope = −0.15, p-value = 0.024, r^2^ = 0.04). In the regression model, five endangered and critically endangered species (solid points on Fig. 2a,b) were included. However, it was also likely that these species were very rare and, as such, their catches obtained in the catch assessment could be misleading. Therefore, when they were dropped from the analysis, the significant relationships were indicated with both maxTL (slope = −0.13, p-value = 0.006, r^2^ = 0.06) and trophic level (slope = −0.16, p-value = 0.02, r^2^ = 0.05).

**Figure 3:**
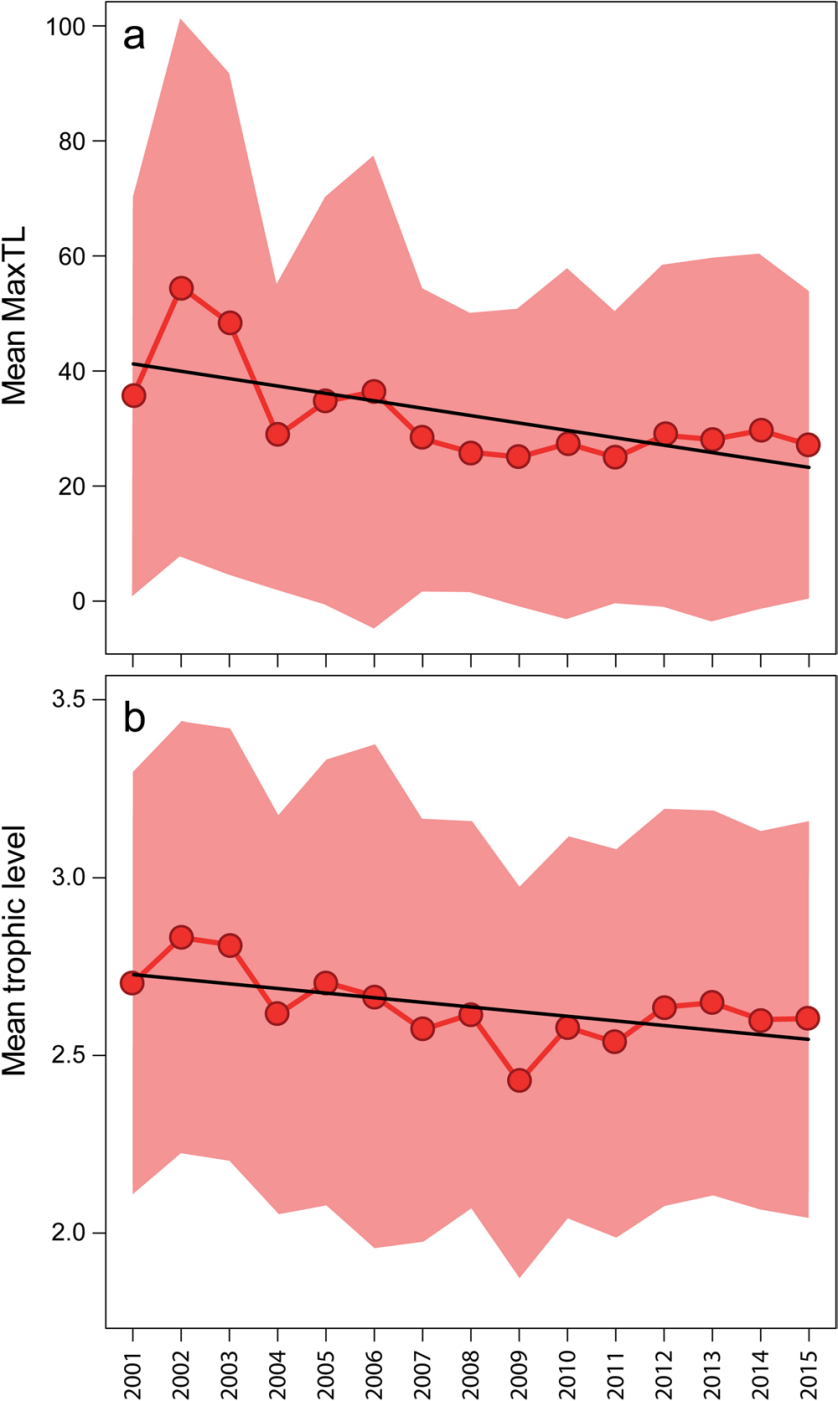
Weighted mean: (**a**) maximum total length (maxTL) and (**b**) trophic level in seasonal catches of the *Dai* fishery from the fishing season of 2000/01 to 2014/15. For model summary (**a**), intercept = 42.53, slope = −1.29, predictor p-value = 0.007, r^2^ = 0.44. For model summary (**b**), intercept = 2.74, slope = −0.013, predictor p-value = 0.025, r^2^ = 0.33. Pink shaded area denotes standard deviation around the mean values. 2001 represents the fishing season of 2000/01 and the same for other years.

When grouped by positive and negative standardized regression coefficient values (for all 116 species), maxTL was significantly greater for the species with negative standardized regression coefficients than the positive ones (Fig. 2c; Mann-Whitney rank sum test, p-value = 0.02). Negative values of standardized coefficients were noted for species with maximum length corresponding to >45 cm (3^rd^ quartile), whereas positive standardized regression coefficients were noted for species with maxTL <25 cm (2^nd^ quartile). Species with both negative and positive coefficient values fell within maxTL of ~25 cm and ~45 cm. Trophic level did not significantly differ between negative and positive standardized regression coefficients (Fig. 2d; Mann-Whitney rank sum test, p-value = 0.08). Nevertheless, species with negative standardized coefficients had higher trophic levels >3.3 (3^rd^ quartile), and species with positive standardized regression coefficients had lower trophic levels (<2.75). Species with both negative and positive coefficient values fell within trophic levels of ~2.75 and ~3.3. Furthermore, weighted mean maxTL and trophic level of seasonal total catch (Fig. 3a,b) oscillated with a mean range of ~25–55 cm and ~2.4–2.8, respectively, and significantly declined across the 15-year period (mean maxTL: slope = −1.26, p-value = 0.007, r^2^ = 0.44; mean trophic level: slope = −0.013, p-value = 0.025, r^2^ = 0.33). Although some small-bodied species including *Parachela siamensis* (maxTL: 18.3 cm; trophic level: 3.4), *Parambassis wolffii* (maxTL: 24.4 cm, trophic level: 3.72) and *Acantopsis* sp. cf. *dialuzona* (maxTL: 30.5, trophic level: 3.5) also exhibited significant declines in seasonal catches (standardized coefficients < −0.66), our combined findings indicate that smaller, lower trophic position species increased and compensated for declines in larger bodied, higher trophic position species in the Tonle Sap fishery over the study period.

### Temporal change in weight and length of individual fish

**Figure 4:**
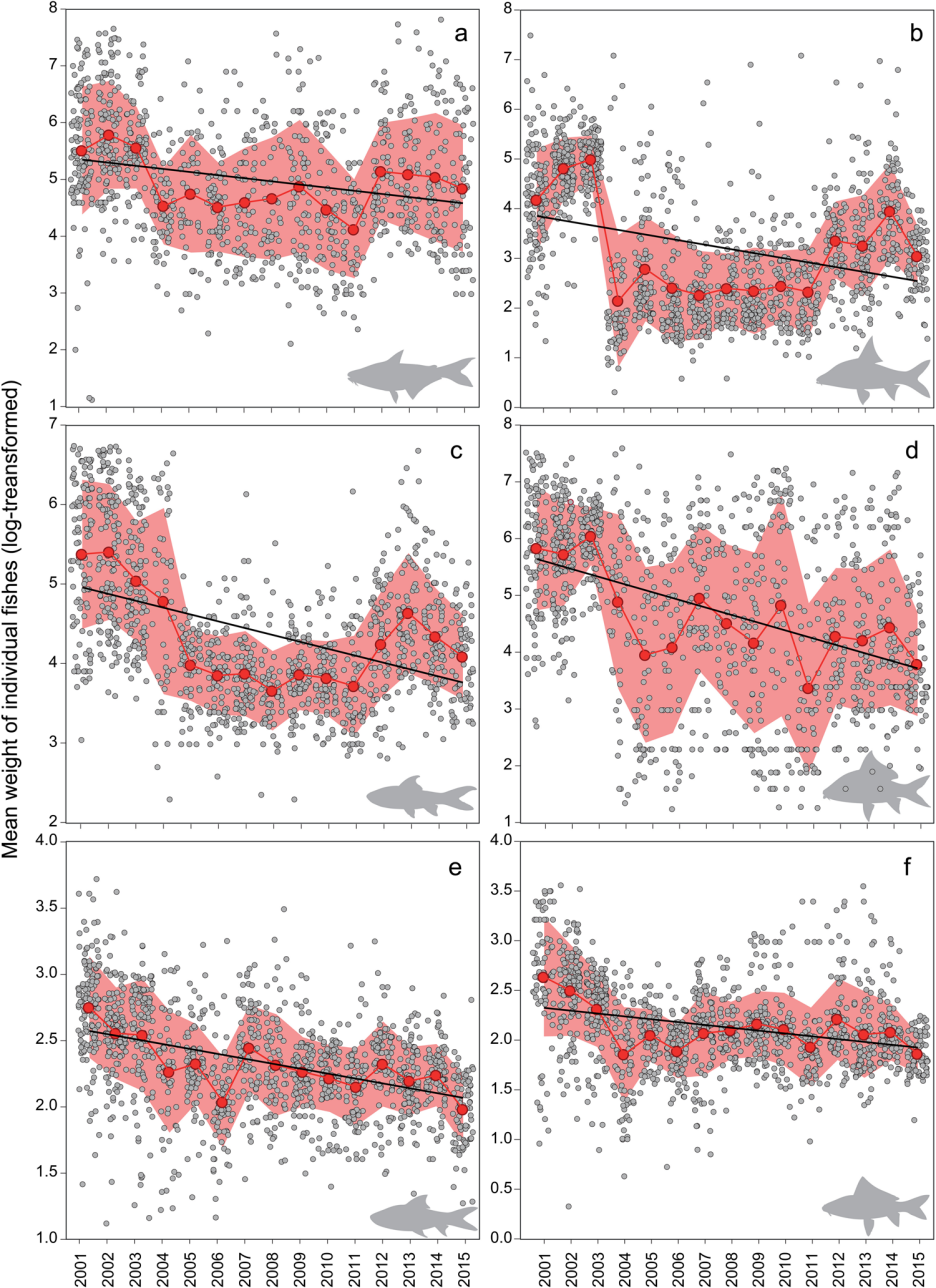
Linear regressions demonstrate temporal change in log-transformed mean individual weight (gram) by season of six common species, composing of large (**a**): *Pangasianodon hypophthalmus,* (**b**): *Cyclocheilichthys enoplos;* medium (**c**): *Cirrhinus microlepis,* (**d**): *Osteochilus melanopleurus* and small-sized species (**e**): *Henicorhynchus lobatus,* (**f**): *Labiobarbus lineatus* that possessed either negative (**a**–**d**) or positive (**e**,**f**) catch changes (expressed as standardized regression coefficients, Table S6) from the fishing season of 2000/01 to 2014/15. See Table S7 for parameter estimates. All slopes were significant (p-value < 0.0001). Solid red dots indicate mean body weight and the pink shaded area denotes standard deviation for each survey season across the study period. 2001 represents the fishing season of 2000/01 and the same for other years.

The log-transformed mean fish body weight captured per day in the *Dai* fishery significantly decreased over the study period for all 6-species explored (p-value < 0.0001; Fig. 4; parameter estimates provided in Table S7). These species span a range in body size (large, medium and small) and regression coefficients indicated that individual fish weight consistently declined through time for all 6 species regardless of body size (Fig. 4a–f).

**Figure 5:**
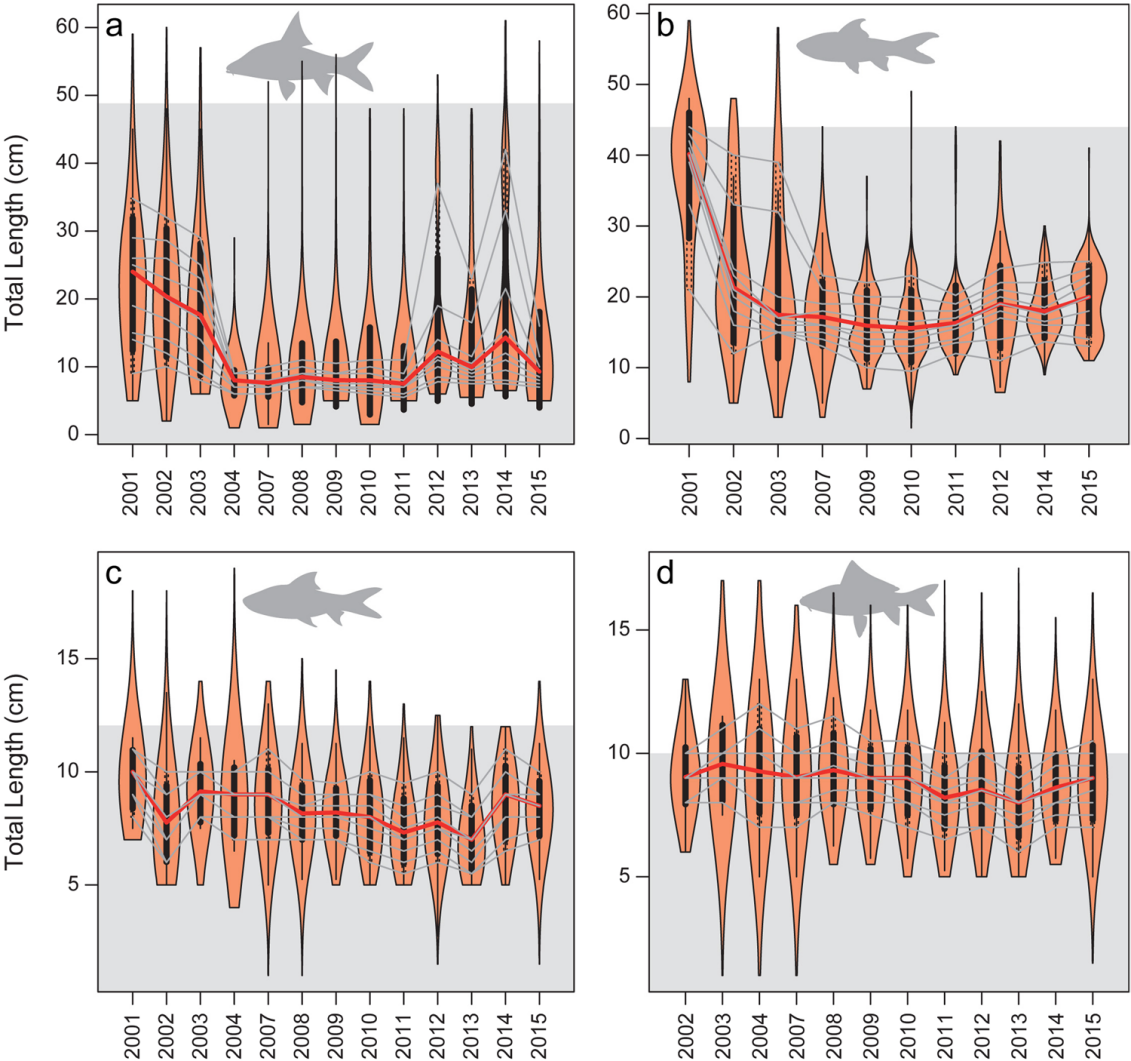
Violin plots show temporal shift in length distribution of four species (**a**): *Cyclocheilichthys enoplos*, (**b**): *Cirrhinus microlepis*; (**c**): *Henicorhynchus lobatus*; (**d**): *Labiobarbus lineatus* from the fishing season of 2000/01 to 2014/15. Red solid line symbolizes median body size in each fishing season and grey thin lines indicate decile, dividing ten equal groups of a population. Area above the gray shaded area denotes estimated total length at maturity for each species. 2001 represents the fishing season of 2000/01 and the same for other years.

Violin plots further elucidated the temporal changes of the total length for four common species (Fig. 5). For the large- and medium-sized species *Cyclocheilichthys enoplos* (maxTL: 90.3 cm) and *Cirrhinus microlepis* (maxTL: 79.3 cm), both of which were mainly captured at juvenile sizes with the average total length <20 cm and 25 cm, respectively; body lengths have declined since the early 2000s when some comparatively large individuals (>30 cm) were present in the *Dai* fishery’s catches (Fig. 5a,b). Noticeably, the medians for these large and medium-sized species were significantly lower than 49 and 44 cm (Fig. 5a,b), the estimated lengths at maturity for *C*. *enoplos* and *C*. *microlepis*, respectively. For the smaller species (maxTL< 20 cm), *H*. *lobatus* and *L*. *lineatus*, which are common and highly productive, total length in the *Dai* catches had a median of ~9 cm, with some individuals possessing lengths greater than lengths at maturity which are ~12 cm for *H*. *lobatus* and ~10 cm for *L*. *lineatus* (Fig. 5c,d). Both species also exhibited gradual decrease in the median total length, but less pronounced than those of large-sized species.

## Discussion

Trends in the seasonal catches of harvested species revealed that 78% of the 116-species are in decline. While we do not have fishery independent data to confirm the large *Dai* dataset, our results are consistent with the prediction of an intensively exploited indiscriminate fishery. Consistent with indiscriminate fishing theory, a closer examination of the data indicated that the catch declines are disproportionally represented by the larger, slower growing, higher trophic level organisms of the Tonle Sap. By contrast, the 22% of species caught by the *Dai* fishery that have tended to show increases are disproportionally represented by small-bodied, faster growing lower trophic level organisms. In addition, significant declines of the mean fish size and trophic level were evidenced in the seasonal catches of the fishery over the study period (Fig. 3a,b). Finally, the data consistently showed for common species spanning a range in adult body sizes that individual weights and lengths of all these species, even in many of the small-bodied species, have been significantly reduced over the last 15 years, a result that resonates with much research that has found that heavy fishing pressure is known to drive shifts in life history towards smaller sizes and earlier ages at maturation^24^. Our results also pointed out for select species that the number of immature fish captured has increased throughout the study period. Moreover, a significant decreasing trend in species evenness was observed over the study period (Fig. S5). Thus, although this fishery has been amazingly resilient to changes in total fish harvest levels, these results collectively are in agreement with predicted effects of indiscriminate fishing theory. Because this theory ultimately predicts declines in fish catches and diversity with sustained, heavy indiscriminate fishing pressure^13–15,25^, our findings may be seen as an ‘early warning signal’ of looming negative impacts of indiscriminate fishing in the Tonle Sap.

Intriguingly, recent work has argued that such indiscriminately fished systems may generally occur in tropical systems where fish is the major source of animal protein^26^. Consistent with this conjecture, recent empirical fisheries data in the East China Sea^15^, where fish is also a major source of protein, has argued that this fishery is relatively indiscriminate and has also showed a compensatory positive growth response by small fish to heavy fishing. Further, and consistent with our results, they argued that this compensatory response helped maintain fishery production^21^. This compensatory response is expected in indiscriminate fisheries as fishing effectively replaces slow growing larger, often higher trophic level fish, with faster growing smaller fish that tend to be from lower trophic levels^13–16^. This reduction of upper trophic level fish drives a cascading effect whereby released predation pressure allows lower trophic level species to flourish^15^. As shown in Fig. S8, CPUE (catch per *Dai* unit per day) in this large fishery fluctuated with no significant trend over the study period suggesting that the smaller fish growth rates are indeed compensating for reduction in upper trophic level catches. Given the reduction in mean body size and trophic level over time (Fig. 3a,b) as well as the average positive growth rates of small species (Fig. 1) our results suggest that small faster growing species are compensating for the heavy fishing pressure.

Our findings of declining catches of medium and large-sized species as well as falling mean body size of fish catches support general perceptions by fishers throughout the Lower Mekong Basin^27–29^. Our results, therefore, are consistent with existing knowledge that some giant- and large-sized fish populations in the Mekong region have declined since the 1900s. For example, the Mekong giant catfish (*Pangasianodon gigas*) (maxTL: 300 cm, max. weight: 350 kg), which was common and abundant in the 1900s, has almost disappeared from the Mekong River System^28,30^. Tonle Sap River is one of the last few places where a small number of individuals of this species are still occasionally captured^30^. In particular, the standardized regression coefficient for this species was almost zero (0.03), indicating little change in its contribution to the *Dai* catch since 2000. This perhaps reflects either effectiveness of conservation measures or that its population status is close to extinction. Likewise, the Mekong giant carp (*Catlocarpio siamensis)* (maxTL: 300 cm, max. weight: 300 kg) was seen regularly in the catch of 1938–39 and 1962–63^31,32^. Nowadays, however, the Mekong giant carp has become critically endangered. Similarly, the Mekong shad (*Tenualosa thibaudeaui*) (maxTL: 60 cm) was still relatively abundant in the *Dai* catch in 1938–39 and 1962–63 and used to be one of the most important species. Nonetheless, it too has been experiencing drastic decline during the last two decades^33^. The list of large-bodied species in decline goes on. Jullien’s golden carp *(Probarbus jullieni)* (maxTL: 183 cm, max. weight: 70 kg) was noticed as ‘comparatively scarce’ for at least 65 years in Thailand^34^, and together with Thicklipped barb *(Probarbus labeamajor)* were later observed to be very abundant in 1970s in the southern Laos and northern Cambodia (when the region was at war). Both have declined, particularly since 1990s when the region’s border trade was re-opened up^22,35^ and now these two species are considered to be endangered by the IUCN Red List. Similarly, other formerly-common and high value species, including *Cirrhinus microlepis* (maxTL: 79.3 cm), have been assessed as a vulnerable species in the IUCN Red List. Based on our analysis, these giant- and large-sized species have all declined during 2000–2015.

The decline in the giant and larger-bodied species is likely associated with their slower growth and late age at maturation. For instance, both *P*. *gigas* and C. *siamensis* do not reach maturity until ~7 years of age^36^. These larger species often require large geographical ranges to complete their life cycles and undertake long migrations between critical habitats^33,37^, making them more susceptible to capture before their first reproductive event. Given the increasing fishing pressure in the region, overfishing seems a likely cause of the decline observed in giant, large and medium sized fishes in the Tonle Sap, which is consistent with previously observed declines in long-lived, late spawning freshwater fish stocks such as the Murray River cod in Australia and ~21 sturgeon stocks across Asia, Europe and America and Pirarucu (*Arapaima gigas*) in Amazon^38^.

In contrast to large-bodied fish, the catch of some small-sized species such as *Labiobarbus* spp. (synonym of *Dangila* spp.) increased significantly over the study period. For instance, members of this genus accounted for ~5% of the *Dai* fishery catches between 1995 and 2000^28^ but increased to 19% in 2013/14 fishing season^39^. Additionally, *Henicorhynchus* spp., which are ecologically important in the LMB^35,40^, made up 25.4% of the total *Dai* catch weight in 1962/63^32^ but increased to 40% between 1995 and 2000^28^ and increased again to 43% in 2013/14 fishing season^39^. Comparable increasing trends are also manifested for other small-sized cyprinids that are likely more robust to fishing pressure and also reproduce quickly on the vast area of seasonal flooded land every year^13,15,27,33^ once predatory pressures of higher trophic level fish^28,33,41^ are reduced.

While our results from the Tonle Sap revealed that overall declining catches were associated with large-bodied species, some small-sized species were also declining. These species feed in higher trophic levels (3.4–3.7) than some giant- and large-sized species such as the Mekong giant catfish (2.3) and Mekong giant carp (2.92) which are detritus and algae feeders. It is also likely that threat status of freshwater fishes was not as clear-cut as that of the marine fishes as evidenced in a study of extinction risk of European freshwater fishes where small-bodied species were most at-risk due to their small geographical ranges^42^. Likewise, when comparing fish body-size distribution under different global extinction risk levels, threats were found to disproportionately occur to both large- and small-sized species^43^. It is likely that further research on individual life history traits may help shed light on reasons of the decline, which is warranted because overfishing is not be the only threat to the Tonle Sap’s fishes.

Moreover, both increased efficiency of fishing gears and increased human population size have likely contributed to declining large-sized species in the Tonle Sap. In Cambodia, the use of monofilament nylon gillnets was to blame for the decline of *C*. *microlepis*, *B*. *microlepis*, *Probarbus* spp., *T*. *thibaudeaui*, *P*. *hypophthalmus*, *Wallago leeri* (maxTL: 150 cm) and Irrawaddy dolphins^22,41,44^ and were considered as a ‘wall-of-death’ for many migrating fishes^29^. The problems caused by these fishing techniques have likely been exacerbated by population growth and the population of countries sharing the Lower Mekong Basin has increased about threefold between 1960 and 2015. Similarly, the Cambodia population has also grown almost threefold with ~85% rural dwellers^45^. Since entry into fishing is free, and fishing gears are very affordable,^29,44^ a combination of forces including rising population along with the lack of other livelihood options, has resulted in millions of people moving into the fishing sector thereby increasing fishing effort and pressure on fish stocks.

Further, hydropower development in the region also poses an increasingly large additional threat to the Mekong fisheries. Numerous hydropower developments loom over the Mekong Basin threatening to alter flows, fragment habitats, block fish migration routes from completing life cycles, degrade water quality and reduce the overall productivity of rivers resulting from nutrients and sediment losses. This is particularly troubling because the migratory species present in the Tonle Sap represent a third^46^ of the total number of fish species in the Mekong Basin from which some 877 fish species have been recorded^9,47^. In Cambodia, migratory species form 63% of catch by weight from Tonle Sap floodplains^28^ and up to 82% from Tonle Sap River (Result section).

The findings in this paper, for the first time, demonstrate evidence that the catches of the large- and medium-sized species in the Tonle Sap are declining while some small-sized, fast-growing species are increasing and contributing to the maintenance of the *Dai* fishery’s overall catches in the past decades. This is akin to other notable indiscriminate fisheries such as that recently noted in the East China Sea where catches consisted of 1-year-old fish and the high exploitation level has been sustained for at least 10 fish generations^15^. This paper further demonstrates that even small-bodied species, so far capable of increasing their production on average, are showing significant reductions in body size with the consequence of an overall reduction in the percentage of mature individuals. This latter result is a warning signal to fisheries managers and conservationists that the species-rich Tonle Sap, so far able to maintain total harvest levels, may be close to its limit. The findings suggest that enhanced protection and conservation efforts are urgently needed to maintain food security in this region.

Fortunately, formal institutions for fisheries protection and conservation in Cambodia are now in place^48^ with restrictions imposed on fishing seasons, gears and geographical areas (fish sanctuaries). Sufficient resource allocation to the sector are therefore necessary to effectively enforce and monitor these fisheries regulations in order to protect and converse the fish biodiversity in the Tonle Sap, with the main aim to (1) let fish spawn at least for the first time before capture, (2) let fish grow and (3) let the mega-spawners live^49^. Tonle Sap River is specifically a natural passageway for many seasonal migratory fishes in the region. Setting appropriate regulations on the basis of known peak seasonal migrations during the inflow and outflow periods that allows some fishes (including endangered species) to pass through the river, would enable some juveniles and broodfishes to complete their life cycles, i.e. accessing the Tonle Sap floodplains and area south of Phnom Penh to feed, and the upstream of the Mekong mainstream and tributaries to seek dry-season refuge and breed^37,50^. Together with maintaining natural flow and hydraulic conditions for the longitudinal and lateral connectivity among these critical habitats that guarantee free migration routes for fishes are highly likely to be key drivers for the sustainability of the Tonle Sap fisheries. Further, the current formal fisheries management regime favors community-based fisheries co-management, where 516 community fisheries (CFis) including 228 in the Tonle Sap floodplains have been established countrywide^51^. Conservation priority should be given to the CFis situated in these key critical fish habitats. By effectively protecting and conserving these areas combined with appropriate hydraulic conditions, some juveniles and broodfishes may be maintained to sustain the seasonal reproduction, recruitment and growth. For future work, it is worth exploring a modelling approach which is able to suggest a management strategy that maximizes the present benefits from the Tonle Sap fishery while maintaining its long-term sustainability e.g.^52^.

## Methods

### Dai fishery

**Figure 6:**
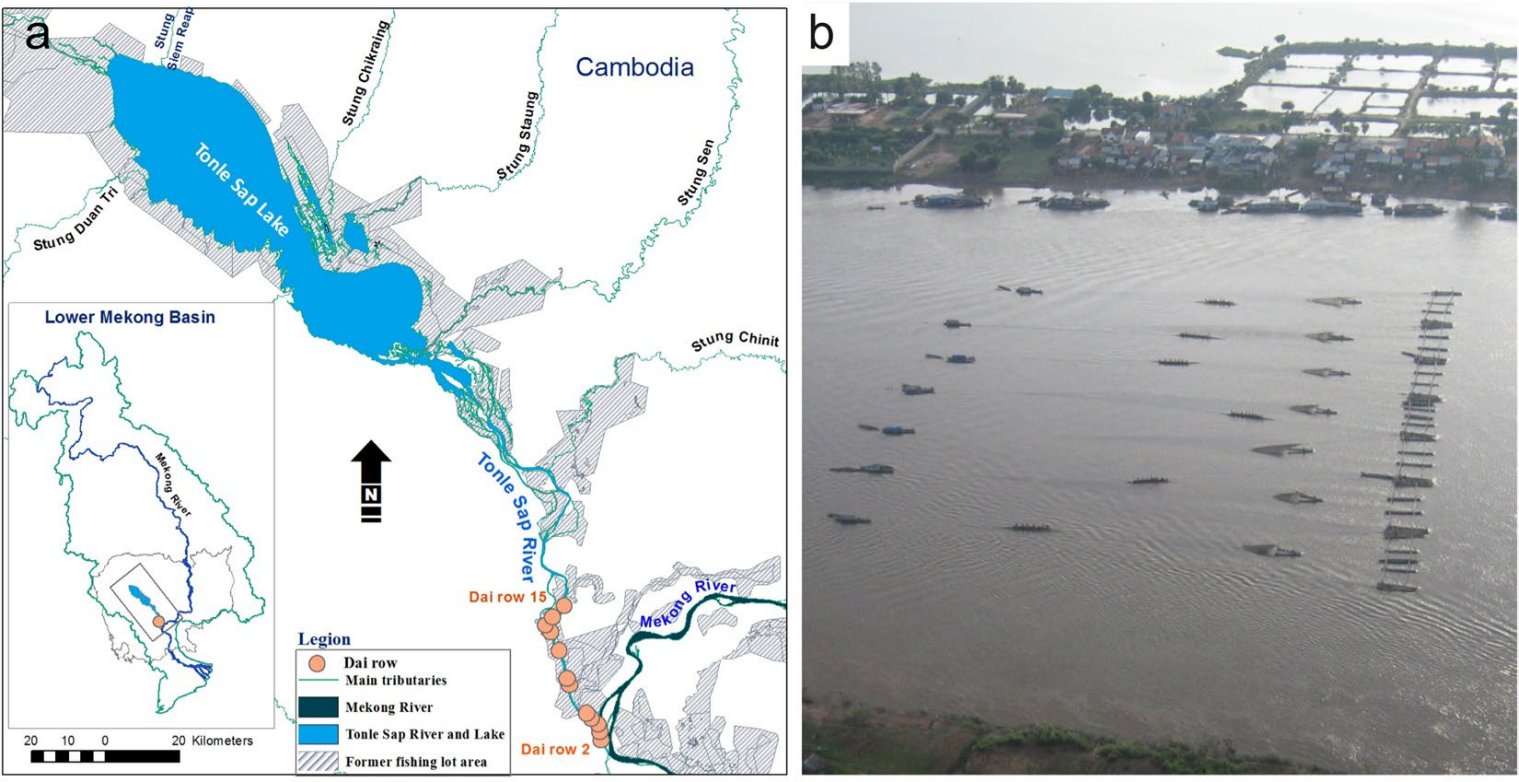
*Dai* fishery in the Tonle Sap River: (**a**) location of *Dais*; (**b**) an aerial photo of a *Dai* row with seven units in operation. Map is created using ArcMap 10.2.2.

This study used time series catch weight data of a standardized assessment on an industrial-scale ‘*Dai* fishery’ between 2000 and 2015 (see also S1). The fishery seasonally operates between October and March in a specific location along the lower section of the Tonle Sap River, stretching about 4–30 km north of Phnom Penh. All *Dai* (64 units) are organized into 14 rows (referred to as row 2 to 15, with the most upstream row 15 situated closest to the Tonle Sap Lake; Fig. 6a) and operated singly or jointly of up to 7 units in a single row (Fig. 6b). A *Dai* unit can be uniquely identified through a combined alpha-numeric code of row number and the letter ‘A’ to ‘H’ of each individual *Dai* in that row. For example, *Dai* 2 A indicates *Dai* A in row number 2. The transversal position of *Dai* rows within the river channel changes along up- and downstream axis (Table S2). In Row 2–4 and 7, *Dai* is positioned towards the right bank (facing upstream) while row 13 and 14 are anchored more to the left bank, and the other units are positioned around the center of the river. Such positions of *Dai* row remain relatively unchanged for more than a century, with the aim to maximize catches dependent on local river morphology and hydrology^53^. Every *Dai* row is never broad enough to block the river, because by law, they have to leave space for navigation^44,48^.

Dai is a relatively indiscriminate fishing gear. The mesh sizes of the gear taper down from ~15 cm at the mouth to 1 cm at the codend. The *Dai* mouth is about ~25 m wide and its opening is determined by the water depth with the lower footrope (with chain) anchored at the river bottom and the upper rope on the water surface. The opening of the *Dai* mouth is maintained by the force of water current. The fishing gear is installed in the Tonle Sap River to filter fish that migrate out of the Tonle Sap floodplains back to the Mekong River during the dry season each year. Overall, the fishing effort of the *Dai* fishery (number of *Dai* units, gear dimensions, season of fishing and geographical location of the fishery) remains relatively constant over the study period, although some increases in hauling time have been reported during the peak migration periods^53^. Technical details of the *Dai* gear are described in^44^. Assuming that (1) the migration of fish from nearby floodplains to the between-*Dai* rows and (2) removals of fish by other small-scale fishing gears operating between *Dai* rows could be ignored, the mean catch rate of the fishery has a general declining slope from row 15 down through row 2 (from closest to furthest away from the Tonle Sap Lake; Fig. 6a), indicating depletion response of fish population which is gradually removed from the system through cumulative *Dai* rows (fishing effort). Each *Dai* unit was predicted to remove 2.8% of migrating fish, and up to 83% of the fish arriving at row 15 were estimated to have captured by the 64 *Dai* units^53^.

### Data collection

Catch data from the *Dai* fishery were made available by the Fisheries Programme of the Mekong River Commission that technically and financially support the catch assessment programme. The catch of the fishery has been routinely assessed by the former Department of Fisheries (currently is the Fisheries Administration - FiA) and later by the Inland Fisheries Research and Development Institute (IFReDI) of the FiA in cooperation with its sub-national counterparts. General concepts and formula for assessing catches and catch composition are outlined in^54^, and these concepts were used to frame the sampling protocols and assessing catches of the fishery. The sampling unit was based on *Dai* unit and a randomly stratified sampling method was used for this paper. More specifically, *Dai* units were stratified based on (Fig. S3): (i) administrative space divided into two strata: Kandal Province (row 15–7 containing 42 *Dai* units) and Phnom Penh Municipality (row 6-2, containing 22 units), (ii) time based on the lunar period: Peak Period occurring in a time-window between 7–1 days before full moon and Low Period, covering the rest of each month for the entire fishing season (iii) *Dai* types: High Catch *Dai* units (11 in Kandal and 6 in Phnom Penh) and Low Catch *Dai* units (31 in Kandal and 16 in Phnom Penh). Relative locations of all *Dai* units within the Tonle Sap River is given in (Table. S2). Sampling on catches per haul or CPUE; including CPUE for species in catch composition and the daily number of hauls of a *Dai* unit were conducted in each stratum, lunar period and *Dai* type within each month of the fishing season for monthly catch estimate. Likewise, fishing effort (number of active *Dai* units and active days) were recorded according to the stratification framework throughout each fishing month over the whole fishing season across the study period. Sampling takes place around 17 days/month with intensive sampling (every day) during the Peak Period and every second or third day in the Low Period.

Catch Per Unit Effort or daily catch rate of the *Dai* unit (kg) is estimated as the product of sampled weight for haul, *i* and estimated number of hauls in a day^53^:

1 $${CPU}{{E}}_{dd,mt,st,lu,dt,dai,i}={weigh}{{t}}_{mt,st,lu,dt,dai,i}.{hau}{{l}}_{dd,mt,st,lu,dt,dai}$$

where *dd* = day, *mt* = month, *st* = stratum, *lu* = lunar period, *dt* = *Dai* type, *dai* = individual *Dai* unit, weight = weight of haul, and haul = estimated number of hauls in a day. Mean daily CPUE is based on mean daily catch samples per haul multiplying by the total number of haul per day. The estimated monthly catch for a given stratum, lunar period and *Dai* type, is as follows:

2 $${Es}.{Mt}.{{C}}_{{mt},{st},{lu},{dt}}={\overline{{CPUE}}}_{{m},{st},{lu},{dt}}\times {Es}.{F}{{E}}_{{mt},{st},{lu},{dt}}$$

where, *Es*.*Mt*.*C* = Estimated Monthly Catch, *Es*.*FE* = Estimated Fishing Effort. Estimated fishing effort is given by:

3 $$Es.F{E}_{mt,st,lu,dt}=A{D}_{m,st,lu,dt}\times A{G}_{mt,st,lu,dt}$$

where *AD* is number of active (fishing) days and *AG* is number of active (fishing) gears for a given stratum, lunar period and *Dai* type. Additionally, estimated monthly species composition is computed as follows:

4 $${Es}.{Mt}.{Specie}{{s}}_{{mt},{st},{lu},{dt}}={SP}{{E}}_{{m},{st},{lu},{dt}}\times {Es}.{Mt}.{{C}}_{{mt},{st},{lu},{dt}}$$

where *Es*.*Mt*.*Species* is Estimated Monthly Catch for a Species, SPE = a fraction of the total estimated catch corresponding to that species and is formulated from the proportion of that species found in the samples. The total catch estimated for a fishing season is the aggregation of the monthly catch estimated for that season.

Apart from sampling data on total catch for each species in each season, data were also obtained for the number, weight and length of some common and commercial individual fish caught per day of each fishing season. These species are among the most ecological, sociocultural (food nutrition and security) and economic important species in the region^33,52,55^. Therefore, they were used to examine the temporal changes in body weight and length for this study (see Figs 4 and 5). Further description of the sample sizes, sampling protocols, data collection forms on catch, species composition and fishing effort, the formula for catch estimation as well as the database system to store and manage the collected data of the fishery are given in detail by^53^.

The current *Dai* fishery database contains information on a total of 141 species. However, only 116 fish species were included in the analysis for this paper because data on the seasonal catches of the other species were sporadic throughout the time series. Furthermore, the species dropped from the analysis were quite marginal in terms of overall catch, and the total catch of the 25-fish species not included in this analysis only represented 0.38% of the total fishery’s catch recorded between 2000 and 2015. Of 116 species, the analysis includes 5 endangered and critically endangered species namely *Probarbus jullieni*, *Probarbus labeamajor*, *Catlocarpio siamensis*, *Pangasianodon gigas* and *Pangasius sanitwongsei*.

In addition to the *Dai* fishery datasets, data were also obtained on maxTL and trophic level of fish species in the Tonle Sap from FishBase^56^. Fish species classification and their ecological attributes were based on the Mekong Fish Database^57^, and are updated using FishBase^56^, in cross-checking with the Catalogue of Fishes Online Database as well as other literature including^58,59^.

### Statistical analysis

All data analyses were performed in R Programme^60^. Linear regression was used to predict the rate of change in the total catch weight of 116 fish species recorded at the *Dai* fishery between 2000 and 2015. The temporal trend for each of the 116 species was expressed as a standardized regression coefficient to allow comparison among species. Standardized regression coefficients measure the change in the dependent variable resulting from a one-standard-deviation change in the independent variable^61^. In univariate linear regression, standardized regression coefficient equals the correlation coefficient (with its values varying between −1 and +1), the intercept equals zero, and the positive and negative signs of standardized coefficients or regression weights (slope) indicates the kind of correlation between the variables^62,63^. Linear regressions, and the generation of standardized regression coefficients, were performed using the ‘lm’ function of ‘stats’ package and ‘lm.beta’ function of ‘QuantPsyc’ package^60^.

Histograms were used to visualize the distribution of standardized regression coefficient values of all species. Simple linear regressions were used to explore the global trend of the relationships between standardized regressions coefficients and species’ maxTL and trophic levels (obtained through FishBase). For all regression analyses, normality was ensured by Shapiro tests (p-value > 0.05). Log +1 transformation was applied to normalize the skewness of standardized regression coefficients prior to the linear regression analyses. In addition, weighted means of maxTL and trophic level in *Dai* catches by season were computed to examine trends of mean maxTL and tropical level across the 15-year study period. To explore temporal trends in the individual weights of the fish constituting the catch, the mean weight of all individuals captured per species per day was calculated and regressed against time. To deal with the data skewness, mean body weight was log-transformed before the analysis, and standard deviation for each species was also computed for each fishing season for the whole study period. This analysis was performed for six common species that spanned a range in standardized regression coefficient values (positive, zero, negative), body sizes and trophic levels, and included large- and medium-sized carps (*Cyclocheilichthys enoplos*, *Osteochilus melanopleurus*, and *Cirrhinus microlepis)*, a large-sized river catfish (*Pangasianodon hypophthalmus)*, as well as small-sized and highly productive mud carps (*Henicorhynchus lobatus* and *Labiobarbus lineatus)*. Being ecological, sociocultural and economic important species, the six species belong to the first two largest families (Cyprinidae and Pangasiidae) forming the largest proportion of the total catches (84% and 4% respectively) from the *Dai* fishery. In addition, *H*. *lobatus* is an ecological keystone species, the most abundant species with its critical role in food security throughout the Lower Mekong Basin (including the Tonle Sap) and an important prey species for many predatory fishes and Irrawaddy dolphins^64,65^. *Labiobarbus lineatus* shares similar ecological characteristics with *H*. *lobatus*. From the *Dai* fishery, *H*. *lobatus* and *L*. *lineatus* are among the most dominant species contributing ~17% and 10% to the *Dai’s* total catch weight respectively. Finally, an attempt was also made to analyze the temporal changes in fish body length of the same six species (as we did with mean body weight). Given that, two of the six species (*P*. *hypophthalmus* and *O*. *melanopleurus)* contained relatively small sample sizes on length, only the other four species were included in the length frequency analysis. Nevertheless, the trends of the four species (Fig. 5a–d) still provide a good example of the status and trends of riverine fishes in the Tonle Sap and Lower Mekong Basin. Length frequency distributions of the four species were then examined across the study period using the ‘violins’ function from ‘caroline’ package in R^66^.

## Electronic supplementary material

Supplementary Information

Data used for this study
