# Supplementary material for: Evidence of indiscriminate fishing effects in one of the world’s largest inland fisheries
Source: Sci Rep. 2018 Jun 12;8:8947. doi: 10.1038/s41598-018-27340-1 (PMC5997758; doi:10.1038/s41598-018-27340-1)
Supplement: Supplementary file 1 — Supplementary Information [file 41598_2018_27340_MOESM1_ESM.doc]

**Title**: Evidence of indiscriminate fishing effects in one of

the world's largest inland fisheries

**Authors:** Peng Bun Ngor^1,2^, Kevin McCann^3^, Gaël Grenouillet^2^, Nam So^4^,

Bailey McMeans^5^, Evan Fraser^3^, Sovan Lek^2^

^1^ Fisheries Administration, No. 186, Preah Norodom Blvd., Khan Chamcar Morn, Phnom Penh, Cambodia

^2^ CNRS, Université Toulouse III Paul Sabatier, ENFA; UMR5174 EDB (Laboratoire Évolution & Diversité Biologique); 118 route de Narbonne, F-31062 Toulouse, France

^3^University of Guelph, Guelph Ontario, Canada

^4^Mekong River Commission

^5^University of Toronto Mississauga, Mississauga, Ontario, Canada

Corresponding author: [pengbun.ngor@gmail.com](mailto:pengbun.ngor@gmail.com)

**Supplementary Information (S)**

**Supplementary information – S1**

***Tonle Sap River System***

The TSRL is a flood pulse system, and is the largest wetland and an integral part of the history, culture, ecology and economics in Southeast Asia ^1^. It is the only continuous area of natural wetland habitats remaining in the Mekong system ^2^. UNESCO approved this area as a world Biosphere Reserve in 1997 ^3^. During the dry season, the lake depth falls to 0.5 meter in late April with a surface area of about 2,000 km^2^ ^4^. During the wet season (June-October), the Tonle Sap River, whose normal flow is from the Tonle Sap Lake to the Mekong River, changes its direction when the Mekong waters rise faster than the Lake. The Lake expands its size four to six times (10,000 to 15,780 km^2^) ^5^, inundating vast terrestrial floodplain areas surrounding TSRL. TSRL’s biological productivity reaches its peak during this period as both migratory fishes from the Mekong and resident fishes in the Lake invade the floodplains for feeding, reproduction and nurseries. Eggs, larvae and fry of fish that spawn upstream in the Mekong mainstream are also carried by the flow and dispersed into the TSRL’s sourrounding floodplains through numerious channels, streams and man-made cannals for feeding, nurseries and growth. When the Mekong flood recedes (September/October) and the Tonle Sap River reverses to its nornal flow, large numbers of fish migrate back to the Tonle Sap Lake, then the Tonle Sap River and Mekong River for dry-season refuges. It is during this period of receeding water (October – March) when *Dai* fishery operates to target these migratory fishes. The fishery usually peaks in December and January in a time window of 6-1 days before full moon during which the river is described as packed solid with fish.

***Dai fishery***

The *Dai* fishery or *Loh Dai*, was established around 140 years ago and resembles a stationary trawl net anchored within the river channel ^1^. At present, it is the only industrial-scale inland fishery remaining in the Lower Mekong Basin (LMB). Catches from the fishery contribute an estimated 14% of the landings from the TSRL system (equivalent to 10% of total fish weight consumed in the LMB), and make up of ~7% to the total inland capture fisheries landings in Cambodia ^1^. The *Dai* fishery seasonally operates in a specific location along the lower section of the Tonle Sap River, stretching about 4-30 km north of Phnom Penh. The river stretch covers two administrative zones: Phnom Penh Municipality and Kandal Province. All *Dai* units are organized into 14 rows and operated singly or jointly of up to 7 units in a single row (Fig. 1). *Dai* row 2-6 are situated in Phnom Penh municipality and row 7-15 are located in Kandal Province with the most upstream row 15 situated in Kandal Province close to the Tonle Sap Lake.

Between the 2000 and 2015 fishing seasons, the number of *Dai* seasonally operating in the Tonle Sap River varied between 60 and 64 units. Generally, a *Dai* unit is between 100 and 120 meters long and 25 meters wide. The net opening (mouth) is determined by the water depth of the river where it is positioned. The size and mesh sizes of the net taper down from the mouth (15 cm) to the cod-end (1 cm). Other details about gear dimensions are technically described by ^6^. *Dai* fishery operation is regulated by a law on Cambodian fisheries ^7^. *Dai* fishery is technically standardized in terms of both location and the gear use which are defined and controlled by the Cambodian law on fisheries. The so-called ‘burden book’, attached to the law, further describes management legislation to be complied by the *Dai* operators. The burden book explains operation rules such as rules on fishing season, *Dai* positions in the river, size restrictions of fishing gear, payment and harvest, detailed descriptions of which are explained by ^1^.

**Supplementary information – S2**

Table S2: Relative *Dai* locations in the Tonle Sap River. The table also indicates sampling stratification scheme which administratively stratifies into Kandal Province and Phnom Penh Municipality. Also, all *Dai* units are stratified into High Catch *Dai* (shaded cells) and Low Catch *Dai* (unshaded cells). The classification of High and Low Catch Dai units was based on the *Dai* catch census, conducted in 1996-1997. Source: adapted from ^1,8,9^.

| Province | Row No. | Approximate  cumulative distance  between rows (km) | Coordinates | | Relative transversal positions of *Dai* nets in the Tonle Sap River | | | | | | | | Total number of *Dai* units forming each row |
| --- | --- | --- | --- | --- | --- | --- | --- | --- | --- | --- | --- | --- | --- |
|  |  |  | North ends | East ends |  |  |  |  |  |  |  |  |  |
| Kandal Province | Row 15 | 37.50 | 11º53.585’ | 104º48.580’ |  | B | C | D | E | F |  |  | 5 |
|  | Row 14 | 33.00 | 11º52.110’ | 104º47.266’ | A | B | C |  |  |  |  |  | 3 |
|  | Row 13 | 31.92 | 11º51.618’ | 104º47.675’ | A |  |  |  |  |  |  |  | 1 |
|  | Row 12 | 28.93 | 11º50.349’ | 104º48.111’ |  | A | B | C | D | E |  | G | 6 |
|  | Row 11 | 23.07 | 11º47.447’ | 104º49.383’ |  | A’ | A | B | C | D |  |  | 5 |
|  | Row 10 | 13.17 | 11º42.257’ | 104º50.515’ |  | A | B | C | D | E | F | G | 7 |
|  | Row 9 | 10.77 | 11º40.963’ | 104º51.026’ |  | B | C | D |  |  |  |  | 3 |
|  | Row 8 | 4.87 | 11º40.477’ | 104º51.360’ |  | B | C | D | E | F | G | H | 7 |
|  | Row 7 | 4.28 | 11º39.685’ | 104º51.969’ |  |  |  | C | D | E | F | G | 5 |
| Sub-Total | 9 rows |  |  |  |  |  |  |  |  |  |  |  | 42 |
| Phnom Penh Municipality | Row 6 | 3.77 | 11º38.867’ | 104º52.581’ |  |  | C | D | E | F | G |  | 5 |
|  | Row 5 | 3.28 | 11º38.363’ | 104º53.328’ |  | B | C | D | E | F |  |  | 5 |
|  | Row 4 | 2.75 | 11º38.295’ | 104º53.809’ |  |  |  |  | A | B | C | D | 4 |
|  | Row 3 | 1.40 | 11º37.640’ | 104º54.705’ |  |  |  |  | A | B | C | D | 4 |
|  | Row 2 | 0.00 | 11º37.068’ | 104º55.116’ |  |  |  |  | A | B | C | D | 4 |
| Sub-total | 5 rows |  |  |  |  |  |  |  |  |  |  |  | 22 |
| Grand total | 15 rows |  |  |  |  |  |  |  |  |  |  |  | 64 |

**Supplementary information – S3**

Fig. S3 Outline of the sampling stratification scheme for the *Dai* fishery catch assessment. The mean catch rate per haul (CPUE) is computed for a *Dai* unit on a day (large shaded area) within each stratum. The total catch is calculated by multiplying the stratum-specific estimate of the mean daily CPUE by the two stratum-specific raising factors: the number of active *Dais* and number of active days ^1^.

*Dai* 1

*Dai* 2

*Dai* 3

High Catch

Peak Period

Low Period

Low Catch

Phnom Penh Municipality

Kandal Province

Month (Total Catch)

Species name

No. of fish

Body weight

Length (selected species)

Big fish sample

Small fish sub-sample

Mean daily Effort per stratum

Total haul

Season (Total Catch)

Mean daily CPUE per stratum

**Supplementary information – S4**

Fig. S4 Relative catch weight (%) of 116 fish species recorded at the *Dai* fishery between 2001 and 2015

**
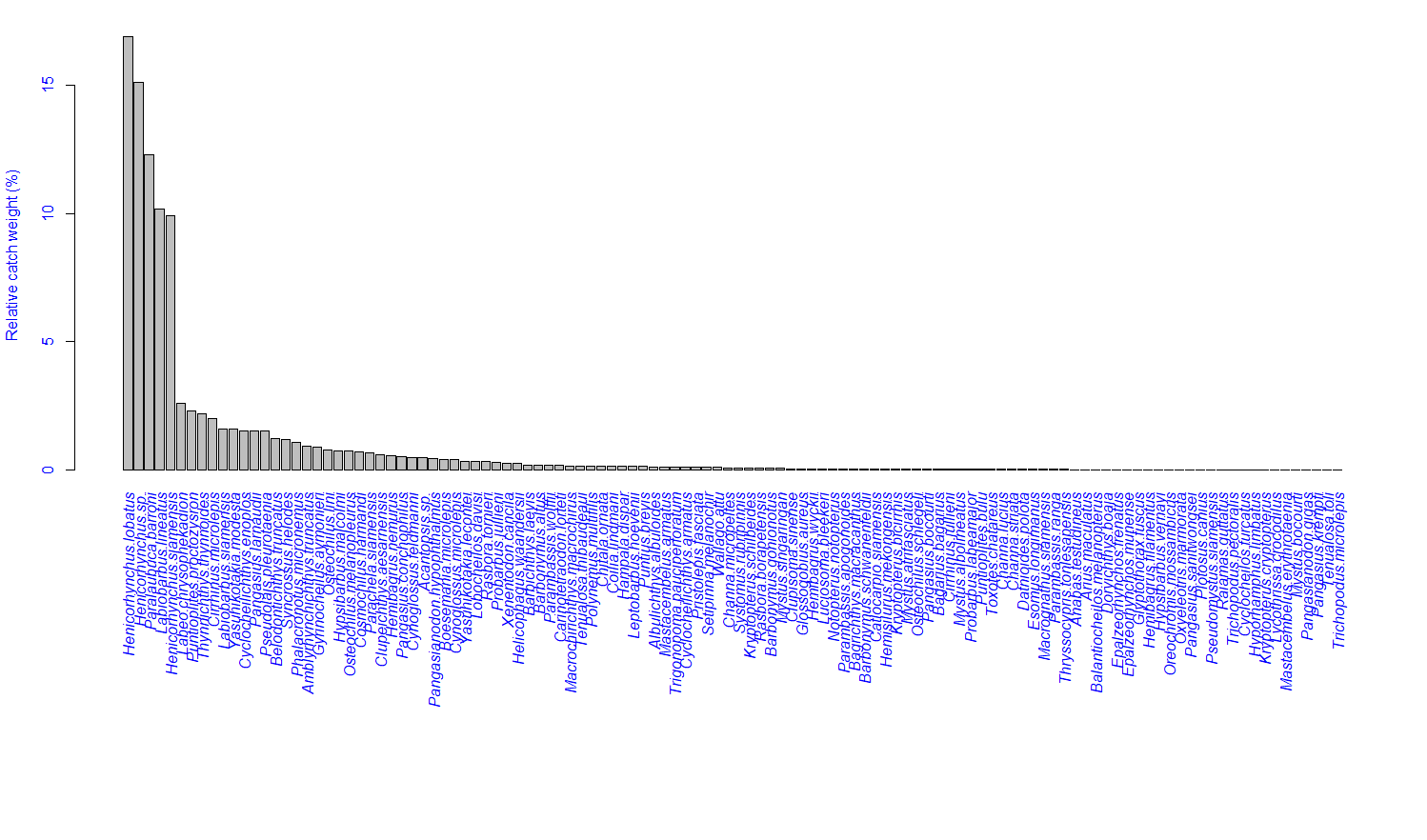
**

Top five species, accounting for 65% of the total catch of *Dai* fishery

**Supplementary information – S5**

Fig. S5 Temporal variations in species evenness recorded at the *Dai* fishery between 2000 and 2015. Species evenness (J) was computed based on **J=H/log(S)**, where H is Shannon diversity index and S is species richness. The value of species evenness varies between 0 and 1, with 0 signifying no evenness and one indicating a complete evenness. Red points are the species evenness values representing fish community for each fishing season. Blue solid line with shaded area around the smooth curve is loess fitting with 95% confidence interval. Overall declining trend of species richness is discerned over the study period between 2001 and 2015.


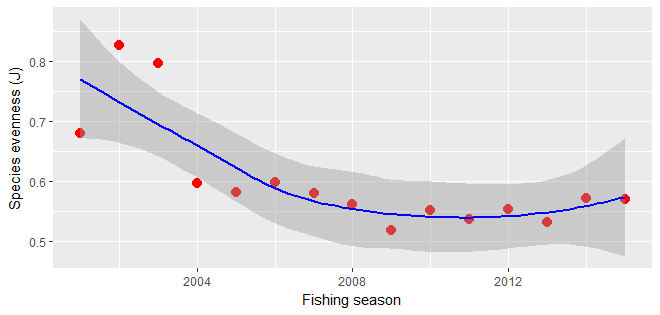


**Supplementary information – S6**:

Table S6: Species’ standardized regression coefficients and ecological attributes

| Species | Standardized  regression  coefficients | Status* | Guild** | maxTL | Trophic level |
| --- | --- | --- | --- | --- | --- |
| *Acantopsis* sp. | -0.68 | ne | 5 | 30.5 | 3.5 |
| *Albulichthys albuloides* | -0.53 | ne | 5 | 36.6 | 2.79 |
| *Amblyrhynchichthys truncatus* | -0.43 | ne | 5 | 48.8 | 2.4 |
| *Anabas testudineus* | 0.21 | ne | 1 | 25 | 2.98 |
| *Arius maculatus* | -0.40 | ne | 5 | 80 | 3.36 |
| *Bagarius bagarius* | -0.53 | ne | 5 | 200 | 3.72 |
| *Bagrichthys macracanthus* | -0.43 | ne | 5 | 30.5 | 2.95 |
| *Balantiocheilos melanopterus* | -0.57 | ne | 5 | 42.7 | 3 |
| *Barbichthys laevis* | -0.26 | ne | 5 | 36.6 | 2.66 |
| *Barbonymus altus* | -0.55 | ne | 3 | 24.4 | 2.4 |
| *Barbonymus gonionotus* | -0.40 | ne | 5 | 40.5 | 2.36 |
| *Barbonymus schwanenfeldii* | -0.47 | ne | 3 | 42.7 | 2.31 |
| *Belodontichthys truncatus* | -0.43 | ne | 5 | 73.2 | 4.08 |
| *Boesemania microlepis* | -0.13 | ne | 3 | 122 | 3.72 |
| *Carinotetraodon lorteti* | 0.15 | ne | 2 | 7.3 | 3.5 |
| *Catlocarpio siamensis* | -0.23 | ce | 5 | 300 | 2.92 |
| *Channa lucius* | -0.35 | ne | 1 | 48.8 | 3.91 |
| *Channa micropeltes* | -0.53 | ne | 1 | 158.6 | 3.85 |
| *Channa striata* | -0.57 | ne | 1 | 122 | 3.36 |
| *Chitala ornata* | -0.56 | ne | 5 | 122 | 3.68 |
| *Cirrhinus jullieni* | 0.56 | ne | 5 | 24.4 | 2.48 |
| *Cirrhinus microlepis* | -0.43 | ne | 5 | 79.3 | 2.38 |
| *Clupeichthys aesarnensis* | 0.25 | ne | 5 | 8.5 | 2.89 |
| *Clupisoma sinense* | -0.03 | ne | 5 | 37.8 | 3.42 |
| *Coilia lindmani* | -0.59 | ne | 2 | 24.4 | 3.74 |
| *Cosmochilus harmandi* | -0.02 | ne | 5 | 100 | 2 |
| *Cyclocheilichthys armatus* | -0.56 | ne | 3 | 26.45 | 3.38 |
| *Cyclocheilichthys enoplos* | -0.69 | ne | 5 | 90.3 | 3.15 |
| *Cyclocheilos furcatus* | -0.30 | ne | 5 | 73 | 3.65 |
| *Cynoglossus feldmanni* | -0.02 | ne | 5 | 30.5 | 3.5 |
| *Cynoglossus microlepis* | -0.60 | ne | 5 | 40 | 3.5 |
| *Datnioides polota* | 0.03 | ne | 2 | 37 | 3.68 |
| *Doryichthys boaja* | 0.49 | ne | 2 | 50 | 3.27 |
| *Epalzeorhynchos frenatus* | 0.53 | ne | 5 | 15 | 2.31 |
| *Epalzeorhynchos munense* | 0.38 | ne | 5 | 11.4 | 2.64 |
| *Esomus longimanus* | -0.56 | ne | 1 | 9.8 | 3.31 |
| *Glossogobius aureus* | -0.32 | ne | 2 | 30.5 | 3.98 |
| *Glyptothorax fuscus* | -0.44 | ne | 5 | 14.8 | 3.2 |
| *Gyrinocheilus aymonieri* | -0.07 | ne | 5 | 34.2 | 2.52 |
| *Hampala dispar* | -0.59 | ne | 5 | 42.7 | 3.7 |
| *Helicophagus waandersii* | -0.60 | ne | 5 | 70 | 3.15 |
| *Hemibagrus filamentus* | -0.38 | ne | 5 | 50 | 3.56 |
| *Hemibagrus nemurus* | -0.35 | ne | 5 | 79.3 | 3.62 |
| *Hemibagrus wyckii* | -0.28 | ne | 5 | 86.6 | 3.76 |
| *Hemisilurus mekongensis* | -0.53 | ne | 5 | 80 | 3.3 |
| *Henicorhynchus lobatus* | 0.24 | ne | 5 | 18.3 | 2.74 |
| *Henicorhynchus siamensis* | -0.06 | ne | 5 | 24.4 | 2 |
| *Henicorhynchus* sp. | 0.20 | ne | 5 | 15 | 2 |
| *Hyporhamphus limbatus* | -0.09 | ne | 2 | 35 | 3.1 |
| *Hypsibarbus malcolmi* | -0.59 | ne | 5 | 61 | 3.2 |
| *Hypsibarbus vernayi* | -0.31 | ne | 5 | 26.4 | 2.99 |
| *Kryptopterus bicirrhis* | -0.15 | ne | 5 | 18.3 | 3.89 |
| *Kryptopterus cryptopterus* | -0.17 | ne | 5 | 17 | 3.8 |
| *Kryptopterus schilbeides* | -0.56 | ne | 5 | 12 | 3.78 |
| *Labeo chrysophekadion* | 0.02 | ne | 5 | 90 | 2 |
| *Labiobarbus lineatus* | 0.66 | ne | 5 | 15.5 | 2.49 |
| *Labiobarbus siamensis* | -0.37 | ne | 5 | 22 | 2.3 |
| *Leptobarbus hoevenii* | -0.36 | ne | 5 | 122 | 2.76 |
| *Lobocheilos davisi* | -0.41 | ne | 5 | 9 | 2 |
| *Luciosoma bleekeri* | -0.35 | ne | 5 | 30.5 | 3.78 |
| *Lycothrissa crocodilus* | -0.07 | ne | 5 | 36.6 | 3.71 |
| *Macrochirichthys macrochirus* | -0.58 | ne | 3 | 100 | 3.7 |
| *Macrognathus siamensis* | -0.55 | ne | 1 | 36.6 | 3.26 |
| *Mastacembelus armatus* | -0.50 | ne | 5 | 34.9 | 2.78 |
| *Mastacembelus erythrotaenia* | -0.17 | ne | 5 | 100 | 2.74 |
| *Mystus albolineatus* | -0.33 | ne | 3 | 42.7 | 3.65 |
| *Mystus atrifasciatus* | -0.12 | ne | 3 | 18.3 | 3.04 |
| *Mystus bocourti* | -0.24 | ne | 3 | 29.3 | 3.5 |
| *Mystus singaringan* | -0.47 | ne | 3 | 36.6 | 3.77 |
| *Notopterus notopterus* | -0.44 | ne | 3 | 73.2 | 3.6 |
| *Oreochromis mossambicus* | 0.45 | ne | 5 | 47.6 | 2.17 |
| *Osteochilus lini* | -0.56 | ne | 5 | 18.3 | 2 |
| *Osteochilus melanopleurus* | -0.78 | ne | 5 | 73.2 | 2.32 |
| *Osteochilus schlegeli* | 0.00 | ne | 3 | 49 | 2 |
| *Oxyeleotris marmorata* | -0.34 | ne | 1 | 79.3 | 3.9 |
| *Pangasianodon gigas* | 0.03 | ce | 5 | 300 | 2.3 |
| *Pangasianodon hypophthalmus* | -0.65 | ne | 5 | 158.6 | 3.12 |
| *Pangasius bocourti* | -0.37 | ne | 5 | 146.4 | 3.18 |
| *Pangasius conchophilus* | -0.35 | ne | 5 | 146.4 | 2.73 |
| *Pangasius krempfi* | -0.13 | ne | 5 | 146.4 | 2 |
| *Pangasius larnaudii* | -0.39 | ne | 5 | 158.6 | 3.26 |
| *Pangasius sanitwongsei* | 0.20 | ce | 5 | 366 | 3.99 |
| *Parachela siamensis* | -0.73 | ne | 3 | 18.3 | 3.42 |
| *Paralaubuca barroni* | 0.06 | ne | 3 | 18.3 | 3.3 |
| *Parambassis apogonoides* | -0.15 | ne | 3 | 12.2 | 2.87 |
| *Parambassis ranga* | -0.01 | ne | 1 | 8 | 3.27 |
| *Parambassis wolffii* | -0.68 | ne | 3 | 24.4 | 3.72 |
| *Phalacronotus micronemus* | -0.46 | ne | 5 | 61 | 4.03 |
| *Plotosus canius* | -0.28 | ne | 2 | 150 | 3.88 |
| *Polynemus multifilis* | -0.41 | ne | 2 | 34.2 | 3.74 |
| *Pristolepis fasciata* | -0.67 | ne | 3 | 20 | 3.19 |
| *Probarbus jullieni* | -0.20 | e | 5 | 183 | 3.17 |
| *Probarbus labeamajor* | -0.12 | e | 5 | 183 | 2.47 |
| *Pseudolais pleurotaenia* | 0.42 | ne | 5 | 42.7 | 2.42 |
| *Pseudomystus siamensis* | -0.44 | ne | 5 | 18.3 | 3.3 |
| *Puntioplites bulu* | 0.14 | ne | 5 | 35 | 2.37 |
| *Puntioplites proctozysron* | -0.35 | ne | 5 | 30 | 2.7 |
| *Puntius brevis* | -0.32 | ne | 3 | 14.6 | 2.91 |
| *Raiamas guttatus* | 0.59 | ne | 5 | 36.6 | 3.89 |
| *Rasbora borapetensis* | 0.55 | ne | 3 | 7.3 | 3.29 |
| *Rasbora tornieri* | -0.49 | ne | 3 | 20.7 | 3.2 |
| *Setipinna melanochir* | -0.39 | ne | 2 | 40.3 | 3.88 |
| *Syncrossus helodes* | 0.28 | ne | 5 | 36.6 | 3.31 |
| *Systomus rubripinnis* | -0.37 | ne | 5 | 30.5 | 2.88 |
| *Tenualosa thibaudeaui* | -0.41 | ne | 5 | 36.6 | 2 |
| *Tenualosa toli* | 0.27 | ne | 5 | 60 | 2.48 |
| *Thryssocypris tonlesapensis* | -0.40 | ne | 2 | 7.8 | 3.2 |
| *Thynnichthys thynnoides* | -0.31 | ne | 3 | 25 | 2.31 |
| *Toxotes chatareus* | 0.03 | ne | 2 | 48.8 | 3.99 |
| *Trichopodus microlepis* | -0.48 | ne | 1 | 16 | 3.36 |
| *Trichopodus pectoralis* | -0.35 | ne | 1 | 25 | 2.76 |
| *Trigonopoma pauciperforatum* | 0.05 | ne | 3 | 7 | 3.3 |
| *Wallago attu* | -0.54 | ne | 5 | 240 | 3.68 |
| *Xenentodon* sp. | 0.02 | ne | 5 | 40 | 3.86 |
| *Yasuhikotakia lecontei* | -0.51 | ne | 5 | 18.3 | 3.41 |
| *Yasuhikotakia modesta* | 0.13 | ne | 5 | 30.5 | 3.4 |

*ne = not endangered, e = endangered, ce = critically endangered, ** 1 = black (resident) species, 2 = estuarine species, 3 = grey (lateral-migration) species, 5= white (longitudinal/riverine-migratory) species. maxTL= Maximum total length (cm).

**Supplementary information – S7**

Table S7. Parameter estimates from Figure 4. All slopes were significant (p-value < 0.0001). Note that mean body weight is log-transformed.

| Fig.4. label | Species name | Intercept | Slope (year) | R^2^ |
| --- | --- | --- | --- | --- |
| a | *Osteochilus melanopleurus* | 283.79 | -0.139 | 0.17 |
| b | *Cyclocheilichthys enoplos* | 190.86 | -0.094 | 0.10 |
| c | *Pangasianodon hypophthalmus* | 108.73 | -0.0517 | 0.05 |
| d | *Cirrhinus microlepis* | 175.85 | -0.085 | 0.17 |
| e | *Henicorhynchus lobatus* | 73.61 | -0.036 | 0.17 |
| f | *Labiobarbus lineatus* | 59.47 | -0.029 | 0.08 |

**Supplementary information – S8**

Fig. S8. Catch (kg) per *Dai* unit per day (log-scale) over the fishing season from 2000/2001 to 2014/2015. Year on the x-axis indicates fishing season. For example, 2001 represents the fishing season of 2000/2001 and the same for other years. The linear trend of the daily catch per *Dai* (against time) is relatively flatlined. Although the slope is negative, it was not significant (p-value = 0.982).


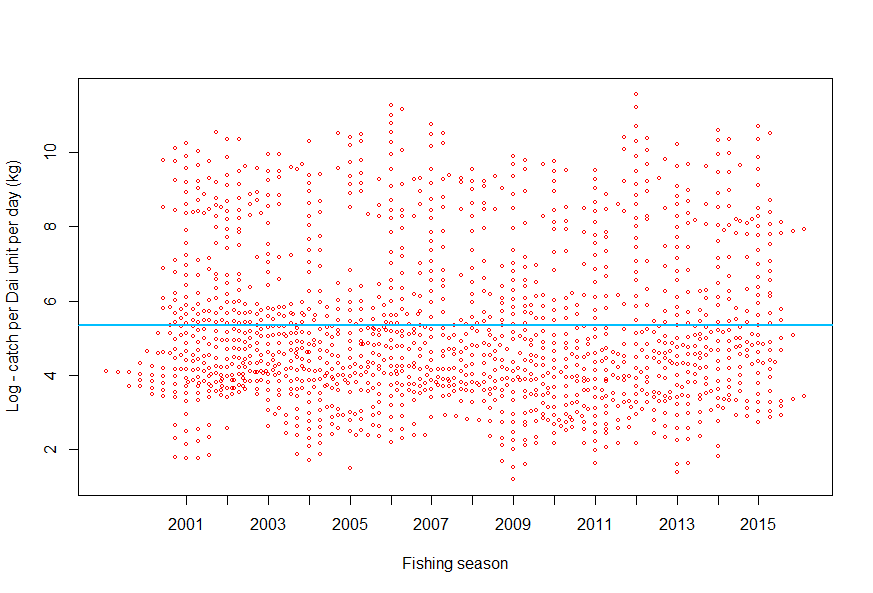


References

1. Halls, A. S. *et al.* *The Stationary Trawl (Dai) Fishery of the Tonle Sap-Great Lake System, Cambodia*. (Mekong River Commission, 2013).

2. van Zalinge, N. *et al.* The Mekong River System. in *Proceedings of the second international symposium on the management of large rivers for fisheries* (eds. Welcomme, R. & Petr, T.) 335–357 (FAO Regional Office for Asia and the Pacific, 2004).

3. Davidson, P. J. *The biodiversity of the Tonle Sap Biosphere Reserve: 2005 status review*. (UNDP-GEF funded Tonle Sap Conservation Project, 2006).

4. MRC. *Mekong: A River for the People*. (Mekong River Commission, 2007).

5. Rainboth, W. J., Vidthayanon, C. & Mai, D. Y. *Fishes of the Greater Mekong Ecosystem with Species List and Photoraphic Atlas*. (Museum of Zoology, University of Michigan, 2012).

6. Deap, L., Degen, P. & van Zalinge, N. *Fishing gears of the Cambodian Mekong*. (Inland Fisheries Research and Development Institute of Cambodia & Mekong River Commission, 2003).

7. FiA. *Law on Fisheries*. (Fisheries Administration, Ministry of Agriculture, Forestry and Fisheries, 2006).

8. Ngor, P. B. & van Zalinge, N. *Dai (Bagnet) fishery: 1994/95-2000/01: catch assessment methodology and results*. (Mekong River Commission/Department of Fisheries/Danida, 2001).

9. Ngor, P. B. Dai fisheries in the Tonle Sap River of Phnom Penh and Kandal province (including a Review of the Census Data of 1996-97). in *Management aspects of Cambodia’s Freshwater Capture Fisheries and Management Implications, Eleven presentation given at the Annual Meeting of the Department of Fisheries of the Ministry of Agriculture, Forestry and Fisheries, 27-27 January 2000* (eds. van Zalinge, N. P., Nao, T. & Lieng, S.) 30–47 (Mekong River Commission and Department of Fisheries, 2000).
